# Left Ventricular Systolic Function Changes During Pump-Assisted Beating Heart Coronary Artery Bypass Graft Surgery: A Prospective Observational Study

**DOI:** 10.7759/cureus.73011

**Published:** 2024-11-04

**Authors:** Ajith BM, Rajesh Raman, Rati Prabha, Dinesh Kaushal, Karan Kaushik, Kumar Rahul, Vivek Tewarson

**Affiliations:** 1 Department of Anesthesiology, Kasturba Medical College, Manipal Academy of Higher Education, Manipal, IND; 2 Department of Anesthesiology, King George's Medical University, Lucknow, IND; 3 Department of Cardiac Anesthesiology, King George's Medical University, Lucknow, IND; 4 Department of Cardiovascular and Thoracic Surgery, King George's Medical University, Lucknow, IND

**Keywords:** coronary artery bypass grafting, left ventricle ejection fraction, left ventricle systolic function, mitral annular plane systolic excursion, pump-assisted beating heart cabg, tissue doppler

## Abstract

Background: There is a lack of information about the left ventricle (LV) systolic function changes during pump-assisted beating heart coronary artery bypass graft surgery (PACAB). This study aimed to study the changes in LV systolic function changes during PACAB.

Methods: In this prospective, single-arm, observational study, 70 patients with American Society of Anesthesiologists physical status III or IV of either sex, aged 40-70 years, scheduled to undergo elective PACAB for isolated ischemic heart disease with EF >30% were included. We excluded patients with pregnancy, pericardial effusion, contraindications to transesophageal echocardiography (TEE), regional wall-motion abnormality of basal LV segments, pericardial effusion, right ventricular dysfunction, bundle branch blocks, and atrial fibrillation. After standard anesthesia induction, patients underwent PACAB. LV ejection fraction (EF), mitral annular plane systolic excursion (MAPSE), and tissue Doppler-derived peak mitral annular systolic velocity (s’) were recorded at various time points of surgery. Change in LV EF during surgery was the primary outcome variable of the study. Secondary outcome variables were complications and changes in MAPSE and s’. A repeated measure analysis of variance was used to compare the changes in LV systolic function at various stages of surgery.

Results: Baseline LV EF was 47.73±9.91%. Compared to baseline, changes in EF during and after the surgery were not statistically and clinically significant. Changes in MAPSE and s’ during the surgery were not statistically significant. Complications included postoperative acute kidney injury, stroke, excess bleeding, and pneumonia.

Conclusion: LV systolic function does not vary significantly during PACAB. However, more extensive randomized trials are required to apply these findings for routine use.

## Introduction

Coronary artery disease (CAD) is one of the leading causes of morbidity and mortality worldwide, frequently necessitating advanced interventions to restore adequate myocardial perfusion and preserve cardiac function [1]. One of the most effective surgical interventions for CAD is coronary artery bypass grafting (CABG), which is typically performed under cardiopulmonary bypass (CPB) with a still and arrested heart [1-3]. However, CPB is associated with several potential complications, including systemic inflammatory response, neurological damage, and adverse effects on various organ systems. The common alternative is off-pump CABG (OPCAB), which can result in hemodynamic instability, reduced cardiac output, and compromised myocardial perfusion, potentially affecting the outcome of the procedure adversely [1,3].

Pump-assisted beating heart CABG (PACAB) is an alternative surgical technique aimed at reducing perioperative complications by decreasing the risks due to hypothermic cardiac arrest and preserving hemodynamic stability [4]. A systemic review evaluated the outcomes of 2040 patients after conventional and PACAB [5]. It was found that PACAB resulted in lower morbidity (low cardiac output syndrome, renal failure, and myocardial infarction) and mortality. Other studies have found PACAB to be a safe and effective alternative to OPCAB, allowing a higher number of distal anastomoses [6]. In a randomized, prospective study comparing PACAB with OPCAB, it was observed that PACAB was associated with a higher number of grafts and higher left ventricle (LV) ejection fraction (EF) without any difference in morbidity and mortality [7]. The benefits of lower mortality and stroke associated with PACAB are also observed in high-risk patients undergoing CABG due to preservation of hemodynamic stability and end-organ perfusion. [8].

LV systolic function is an important component of intraoperative hemodynamic management of patients undergoing CABG [9-11]. Intraoperative transesophageal echocardiography (TEE) has become an invaluable tool for real-time monitoring of LV systolic function during CABG. TEE provides detailed imaging of the heart, allowing for intraoperative assessment of LV performance using various parameters such as EF, tissue Doppler-derived peak mitral annular systolic velocity (s’), and mitral annular plane systolic excursion of the lateral mitral annulus (MAPSE). During CABG, especially when performed on a beating heart, maintaining optimal LV systolic function is crucial to ensure adequate cardiac output and tissue perfusion [10]. The challenges of performing CABG on a beating heart make it essential to closely monitor LV function to detect and manage any hemodynamic instability promptly. The rationale for conducting this study is based on the need for a deeper understanding of the effects of PACAB on LV function. Although PACAB has been proposed as a method to combine the benefits of OPCAB and traditional CABG, there is a lack of prospective observational data examining its effects on LV systolic function during surgery. The aim of this study was to evaluate LV systolic function during various stages of PACAB.

## Materials and methods

This prospective, observational study was conducted after obtaining ethics committee approval (King George's Medical University, Lucknow; 1392/Ethics/2020) and registration with the Clinical Trials Registry - India (CTRI/2021/03/032240). The study was conducted from January 10, 2021, to December 15, 2021, at a tertiary care hospital after obtaining written and informed consent from all the study participants before their inclusion in the study. All procedures adhered to the ethical standards outlined in the Declaration of Helsinki. We included 70 patients of the American Society of Anesthesiologists physical status III or IV of either sex, aged 40-70 years, scheduled to undergo elective PACAB for isolated ischemic heart disease with EF >30%. We excluded patients with pregnancy, pericardial effusion, contraindications to TEE, regional wall-motion abnormality of basal LV segments, pericardial effusion, right ventricular dysfunction, bundle branch blocks, atrial fibrillation, and refusal to give consent.

After the patient arrived in the operating room, monitors, including an electrocardiogram, pulse oximeter, and non-invasive blood pressure, were applied. Intravenous (IV) 0.5 µg/kg fentanyl was administered, and an arterial catheter was inserted in the right radial artery under local anesthesia. General anesthesia induction was done according to the department’s anesthesia protocol using IV etomidate, fentanyl, and vecuronium. This was followed by maintenance with sevoflurane, vecuronium, fentanyl, and midazolam. A central venous line was inserted in the right internal jugular vein, and an arterial catheter was inserted in the femoral artery after induction of anesthesia. A TEE probe was inserted after induction for assessment of intraoperative LV function. Echocardiographic measurements were done using Mylab X8 (Esaote S.P.A, Genoa, Italy) ultrasound machine with phase-array 3-8 MHz TEE probe for intraoperative and 1-5 MHz transthoracic echocardiography (TTE) probe for preoperative and postoperative measurements.

The surgical approach included median sternotomy and partial bypass. The cannulation for the pump was achieved using the right atrium and ascending aorta. PACAB was performed using mechanical circulatory support, with a pump maintaining systemic perfusion without cardiac arrest and aortic cross-clamping. Normothermia was maintained at approximately 36 degrees Celsius throughout the surgical duration. After completion of the surgery, the patients were shifted to the postoperative ICU without extubation. Patients were followed up till discharge from the hospital. The primary outcome variable was change in LV EF at various stages of surgery. The secondary outcome variables were changes in LV function measured by MAPSE and s’ and complications.

EF using the modified Simpson biplane method was measured one day before surgery (baseline) and one day after surgery using TTE [12]. TEE was used for measuring EF after induction of anesthesia, sternotomy, pericardiotomy, cannulation, decannulation, and sternal closure [13]. MAPSE and s’ were used for LV function assessment using TEE after induction, sternotomy, pericardiotomy, cannulation, after decannulation, and sternal closure [14-16]. All the measurements were done by a single anesthesiologist with a 10-year experience of doing intraoperative echocardiography. The measurements were taken three times and averaged to get the final value. The patients’ demographic and baseline data and complications were also recorded.

Data was analyzed using IBM SPSS Statistics for Windows, version 23 (IBM Corp., Armonk, NY, USA). Data is presented as mean±SD or number (percentage) as needed. Repeated measure ANOVA was used to compare the change in LV systolic function at various stages of surgery. Complications were compared using χ2 test. A two-sided p<0.05 was considered significant for all the statistical tests. The study had a power of 0.8 and a type I error of 0.05. In a previous trial, the standard deviation of EF in patients undergoing PACAB was 13.8% [17]. To detect a change of 5% in EF during the surgery, a minimum of 62 patients were needed. To compensate for patient exclusions, we included 70 patients in the study. G*Power (Heinrich-Heine-Universität, Düsseldorf, Germany) software version 3.1.9.7 was used to calculate the sample size.

## Results

**Figure 1:**
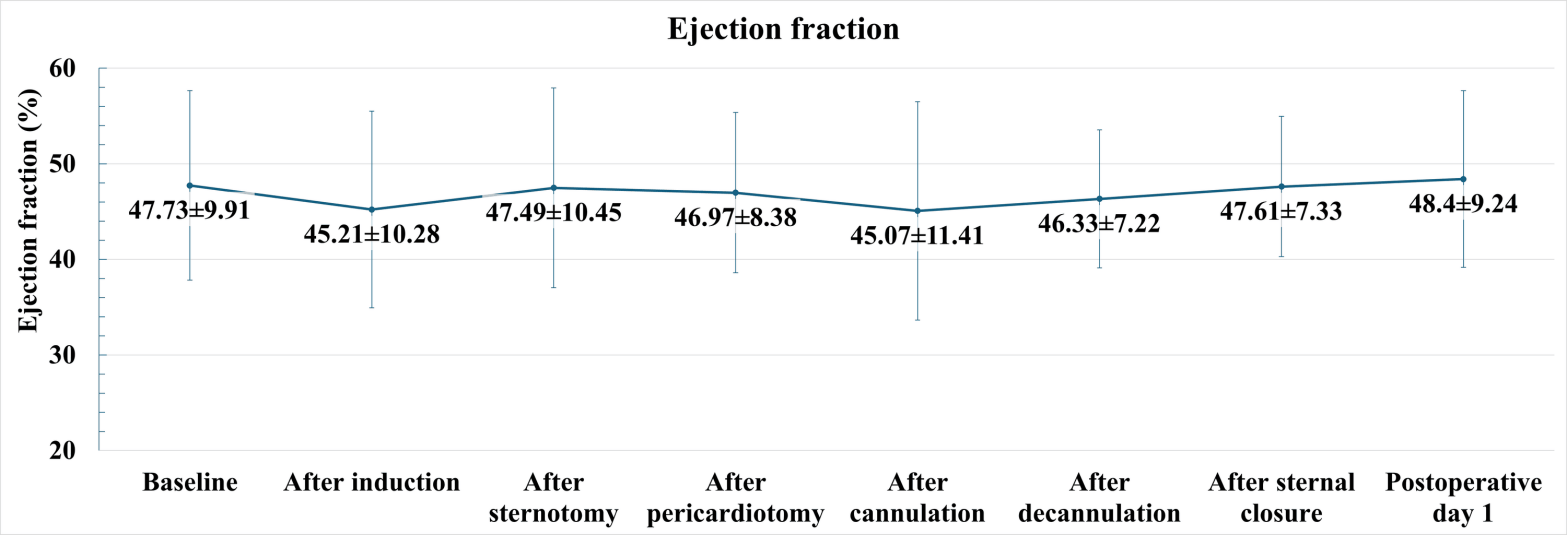
Comparison of ejection fraction at the various stages (n=70, p=0.078, F=2.59).

Out of 123 patients screened for eligibility, 70 patients were recruited in the study. All 70 patients were included in the final analysis of the study. The baseline characteristics of the patients are shown in Table 1. The mean age of the patients was 60.7±8.94 years, with a mean weight of 74.56±14.56 kg. The LV EF at various stages of PACAB is shown in Figure 1. The baseline EF before surgery was 47.73±9.91%. There was no statistically significant change in the EF of the patients after induction of anesthesia, sternotomy, pericardiotomy, cannulation, decannulation, and sternal closure (p=0.078). The EF increased by a small percentage one day after surgery, but the difference from baseline was not statistically significant.

**Table 1:** Baseline and demographic characteristics of the patients. DM: diabetes mellitus, HTN: hypertension, NYHA: New York Heart Association classification, ASA: American Society of Anesthesiologists physical status classification, CBP: cardiopulmonary bypass, M: male, F: female.

Variable	n=70
Age (years)	60.7±8.94
Weight (kg)	74.56±14.56
Height (cm)	166.94±7.91
Male	43(61.43%)
Female	27(38.57%)
NYHA 2	31(44.29%)
NYHA 3	25(35.71%)
NYHA 4	14(20.00%)
ASA III	44(62.86%)
ASA IV	26(37.14%)
DM	47(67.14%)
HTN	38(54.29%)
Smoker	41(58.57%)
No. of Grafts: 2	7(10.00%)
No. of Grafts: 3	55(78.57%)
No. of Grafts: 4	8(11.43%)
Duration of surgery (minutes)	142.07±12.48
CPB duration (minutes)	95.27±10.48

MAPSE and s’ at the various stages of surgery are compared in Table 2. Baseline MAPSE was 11.06±1.48 cm, and s’ was 7.1±1.16 cm-s, indicating mild dysfunction of the LV systolic function. There was no significant change in LV function as measured using MAPSE and s’ from baseline at any stage of surgery. Acute kidney injury and stroke were observed in one (1.4%) patient each. Three (4.3%) patients had excess bleeding, and two (2.9%) patients had postoperative pneumonia (Table 3).

**Table 2:** Changes in MAPSE and s’ at the various stages of surgery (n=70). MAPSE: mitral annular plane systolic velocity, s’: tissue Doppler-derived peak mitral annular systolic velocity.

	After Induction	After sternotomy	After pericardiotomy	After cannulation	After decannulation	After sternal closure	F	p
MAPSE (cm)	11.06±1.48	10.96±1.55	10.95±1.63	10.85±1.5	10.9±1.62	10.91±1.52	0.25	0.86
s' (cm-s)	7.1±1.16	7.04±1.16	6.86±0.91	6.87±0.92	6.88±0.85	7.21±1.00	2.41	0.07

**Table 3:** Frequency of complications in the study (n=70).

Complication	Frequency
Acute kidney injury	1(1.4%)
Stroke	1(1.4%)
Postoperative pneumonia	2 (2.9%)
Excess bleeding	3 (4.3%)

## Discussion

The present study aimed to evaluate LV systolic function during PACAB. The LV systolic function was assessed using EF, MAPSE, and s’ at various stages of surgery. The results indicated that no significant changes in LV systolic function occurred during the various stages of the procedure. The frequency of complications was low in the study. These findings underscore the potential benefits of PACAB in maintaining LV systolic function during CABG.

While systemic perfusion during CABG can be easily achieved using complete CPB, it has its own disadvantages, including the risk of postoperative stroke and neurocognitive defects due to manipulation of the aorta during cross-clamping, systemic inflammatory response, and coagulation defects [18]. The advantages of on-pump CABG include a greater number of grafts and a lesser need for repeat revascularization 30 days after surgery [18]. OPCAB has advantages like lower systemic inflammation and less aortic manipulation. PACAB aims to have the advantages of the two CABG techniques, providing hemodynamic stability using partial CPB and avoiding aortic cross-clamping and hypothermia. By providing partial CPB, the procedure supports the heart during critical phases, thereby maintaining coronary perfusion and reducing myocardial injury [19]. This is particularly important during grafting to anatomically challenging sites such as the posterior or lateral walls, where OPCAB may induce hemodynamic instability due to excessive heart manipulation. Previous studies have reported that OPCAB, although beneficial in avoiding full CPB, can be technically challenging, especially when multiple vessels need to be bypassed [19,20].

LV systolic function is an important determinant of overall cardiac performance, representing the heart's ability to supply blood to systemic and coronary circulation. LV dysfunction during or after cardiac surgery can lead to serious complications, including hemodynamic instability, low cardiac output syndrome, arrhythmias, myocardial injury, and renal dysfunction [21]. Therefore, careful monitoring and management of LV systolic function is important during CABG. However, we could not find trials studying the intraoperative variations of LV systolic function during PACAB.

We chose to evaluate EF as an indicator of LV function as it is the most commonly used and validated method for the assessment of LV systolic function [22]. It is also included in the guidelines for diagnosis and management of LV dysfunction [23,24]. However, baseline and postoperative measurements were taken using TTE, while the intraoperative measurements were taken using TEE. Intraoperative LV function was also assessed using MAPSE and s’ in our study. Both the parameters are highly sensitive and reproducible indicators of LV systolic function and were used to assess the LV systolic function at six time points during surgery using TEE [14-16].

Our study found that the variations in LV systolic function were not significant, both clinically and statistically. The change in EF from baseline in the intraoperative period was less than 3% in our study. As mentioned earlier, we do not have a similar study on PACAB to compare our results. In a trial evaluating the changes in the LV function using EF in patients undergoing OPCAB, it was found that there was a significant change in both the systolic and diastolic function of the heart [10]. The decline in the LV function was observed to be greatest during the grafting of the left circumflex artery and lowest during the grafting of the left anterior descending artery. The LV function and hemodynamic changes returned to normal at the end of the surgery, and no statistical difference could be found at the end of surgery from baseline. However, there are fundamental differences in the surgical and perfusion techniques of the two studies leading to these differences from our study. Our study was done in patients undergoing CABG with the assistance of partial CBP, while in the author’s trial, the patients underwent CABG without any help from CPB. Moreover, the time points of observation were different in the studies. The only time point common between the two studies is the end of surgeries, where there was no difference in LV function from baseline in both studies.

In another study on patients undergoing elective CABG with complete CPB with hypothermia, the change in LV systolic function using EF was studied [9]. It was observed by the authors that there was a significant decline in EF from baseline (52.2±11.2%) to induction of anesthesia (49.8±11.5%). The EF at five minutes and 30 minutes after weaning from CPB was not statistically different from the baseline. The lower EF after the induction of anesthesia was probably due to the use of IV thiopentone sodium, a myocardial depressant drug, for induction of anesthesia by the authors in their study [25]. We did not observe any significant decline in EF in our patients as we used etomidate, a cardio-stable drug, in our study. This avoided any drug-induced depression of the myocardium and preserved EF in our patients.

Our study had a low frequency of complications. Complications included acute kidney injury, stroke, postoperative pneumonia, and excess bleeding. Overall the incidence of complications is similar to those found in a study by Erkut et al. [4]. However, in another study, the frequency of complications was much higher (65%) than that observed in our study [17]. This difference can be due longer period of follow-up in the author's study. The authors included complications up to 30 days after surgery, which was greater than our follow-up period.

The study had two limitations. First, the study was observational and single arm, which may not reflect all the variables encountered in routine clinical practice. Second, we excluded patients with EF<30% in our study, which may limit the generalizability of the study findings to these patients undergoing CABG.

## Conclusions

We evaluated the LV systolic function of patients undergoing PACAB using EF, MAPSE, and s’. It was observed that the changes in LV systolic function during PACAB were not significant clinically and statistically. This study demonstrates that LV systolic function remains stable during PACAB, suggesting that partial CPB effectively supports cardiac performance. These findings highlight the potential advantages of PACAB as a hybrid approach that combines the benefits of traditional on-pump and off-pump CABG. We conclude that LV systolic function does not vary significantly during PACAB. However, more extensive randomized trials are required to apply these findings for routine use.
